# Dr. Bright's Reports of Medical Cases in Guy's Hospital

**Published:** 1828-01-01

**Authors:** 


### VIII.

*Reports of Medical Cases, selected with the View of illustrating the Symptoms and Cure of Diseases, by a Reference to Morbid Anatomy.* By RICHARD BRIGHT, M.D. F.R.S. &c. Lecturer on the Practice of Medicine, and one of the Physicians to Guy's Hospital. Quarto, pp. 231, with 15 Plates, coloured, including a great number of Figures. Longman's, 1827. Price four guineas, boards.

THIS is, beyond all comparison, the most splendid production which this country has ever given rise to, in regard to morbid anatomy. The plates of Baillie, Farre, Hooper, Willan, Bateman, &c. &c. shrink into comparative insignificance, as to accuracy of delineation and beauty of execution, when placed along side of those now before us. The task which Dr. Bright has imposed on himself (for this is only the first of a series of volumes) is truly Herculean, both as respects the labour and the expense. The undertaking is national; for if the author continues " equis passibus," he will not only immortalise himself, but reflect honour on his country—and especially on his own profession. From our Government we cannot—or, at all events, we need not expect, that any reward, honorary or pecuniary, will flow for such meritorious works as this of Dr. Bright; but we do think that if the *learned heads* of departments in our profession obeyed the dictates of *zealous hearts*, they would hold out encouragement for enterprises like this, by conferring some mark of distinction on those who accomplish them. As the production of a physician, our Royal College, we think, should testify its approbation of such a work, even although emanating from an unfortunate Licentiate! It certainly would seem better in the eyes of foreigners to expend a few pounds annually in such a way, than in litigations with the graduates of Scotch universities. We fear, however, that Dr. Bright has little to hope from any other patronage than that of the PUBLIC—a patronage not yet totally swallowed up by the insatiable stomachs of medical monopolists!*

Our author modestly states it as his wish to render, through this publication, the labours of an hospital more permanently useful, by bringing together such facts as seem to throw light upon each other—and, also, to preserve and explain, by faithful engravings, the recent appearances of those morbid changes of structure which have been connected with the symptoms, or have influenced the treatment of the disease.† He considers it unnecessary, in the present day, to expatiate on the utility of hospital reports, or "the importance of that information which our profession derives from the study of morbid anatomy." Dr. Bright must know, however, that it is not many months since discredit was attempted to be thrown on pathology from "high authority," as it has been called—probably, in reference to the geographical position or *altitude* of the theatre where the *sublime* dogmas were delivered. But we must proceed to the work.

This first volume is divided into several sections, the chief of these exhibiting a collection of cases, with appropriate drawings, of the appearances observable in diseases terminating in dropsical effusion:—*first*, of the appearances in the kidney—*secondly*, of those in the liver—and, *thirdly*, of those in the thoracic viscera. There are some other sections on the effects

---

* We think that every opulent individual, and every medical society, should make a point of subscribing to Dr. Bright's book, as the proper way to lend their separate and collective aid in rewarding merit, and promoting the publication of valuable works. There will still be a large class who must take the matter at second hand.

† In passing a high encomium on Dr. Bright's plates, we do not mean to say that they are faultless, or that they are equally meritorious. Those representing diseases of the kidney we consider as the best—and those of the lungs the worst. We think it quite impossible that any portion of lung in phthisis pulmonalis could *faithfully* present all the most brilliant colours of the rainbow, as seen in Plates IX. and X. The same observation would possibly apply to some plates of morbid conditions of the intestines. The second figure in Plate XII. we cannot but think is meretriciously set forth, and as—"flaunting in rags, or fluttering in brocade." The principal, perhaps the whole failing in these beautiful plates, is their *excess of beauty*.

of inflammation in different textures of the lungs—on phthisis pulmonalis—on jaundice—on dysentery—on fever. All these sections we shall notice seriatim, in this and succeeding numbers of our Journal.

### I. ON THE APPEARANCES OBSERVABLE IN DISEASES TERMINATING IN DROPSICAL EFFUSION.

These are exceedingly numerous as well as various—and it is often difficult to say how far these changes of structure are the causes, the auxiliaries, or merely the consequences, of the effusion. One great cause is, unquestionably, obstructed circulation, especially in the venous system. Thus, whatever either generally or locally checks the return of blood through that system, as diseases of the heart, the liver, or the lungs, has a strong tendency to produce serous effusion, either in the cavities or in the cellular tissues. But there are many other diseases besides these more obvious ones of the three great organs alluded to, which give rise to dropsy. Certain affections of the peritoneum, as tuberculation, false membranes, &c. give the tendency to effusion, and leave open a considerable field for the investigations of the pathologist.

Dr. Bright, however, has particularly directed his attention to a class of organic changes which have hitherto attracted too little attention—namely, morbid changes in the kidney, which, "whether they are to be considered as the cause of the dropsical effusion, or as the consequence of some other disease, cannot be unimportant." In these conditions of the kidney, Dr. B. has often found the dropsy connected with the secretion of albuminous urine, more or less coagulable by heat. In such cases, the liver did not betray *any considerable* marks of disease, either in its function during life, or structure after death. On the other hand, where the dropsy has clearly depended on organic disease of the liver, there was generally no morbid alteration in the kidney—no coagulable urine. *Dr. B. avers that he has never found the kidneys free from disease in the bodies of those who have died of dropsy, attended with coagulable urine.* Whether this morbid structure, in its incipient state, may be considered as giving rise to the altered secretion —or whether it be owing to the long-continued disorder of the renal function, may admit of some doubt. Dr. B. is of opinion that the altered action of the kidney is the result of various hurtful causes operating through the medium of the stomach and skin, thus deranging the healthy balance of the circulation, or inducing an inflammatory state of the kidney itself—and that a long continuance of this disturbed function leads to permanent change of structure. This, we think, is the more probable solution; and it is, in fact, in accordance with what we see in other organs of the body, as well as in the kidney. Dr. Bright's observations on the condition of the urine in dropsy coincide, in a great degree, with those of Dr. Blackall. We must observe, however, that Dr. Blackall's conclusions have not been borne out by the experiments of others—and especially by those of Dr. Crampton of Dublin, as seen in the Dublin Hospital Reports.

"Where anasarca has come on from exposure to cold, or from some accidental excess, I have in general found the urine to be coagulable by heat. The coagulation is in different degrees: it likewise differs somewhat in its character: most commonly when the urine has been exposed to the heat of a candle in a spoon, before it rises quite to the boiling point it becomes clouded, sometimes simply opalescent, at other times almost milky, beginning at the edges of the spoon and quickly meeting in the middle. In a short time the coagulating particles break up into a flocculent or a curdled form, and the quantity of this flocculent matter varies from a quantity scarcely perceptible floating in the fluid, to so much as converts the whole into the appearance of curdled milk. Sometimes it rises to the surface in the form of a fine scum, which still remains after the boiled fluid has completely cooled. There is another form of coagulable urine, which in my experience has been much more rare; when the urine on being exposed to heat assumes a gelatinous appearance, as if a certain quantity of isinglass had been dissolved in water. I have indeed met with this in one or two cases only." 3.

In the progress of these anasarcous cases, Dr. B. has generally found a strong tendency to throw off the red particles of the blood by the kidneys, in the form of hæmaturia, varying from the simple dingy colour of the urine, with slight brown deposit, to complete bloody urine, with, occasionally, a thick ropy deposition at the bottom of the pot.

"Besides these cases of sudden anasarcous swelling being generally accompanied by coagulable urine, I have found another and apparently a very opposite state of the system prone to a secretion of the same character; namely, in persons who have been long the subjects of anasarca recurring again and again, worn out and cachectic in their whole frame and appearance, and usually persons addicted to an irregular life and to the use of spirituous liquors. In these cases the albuminous matter has coagulated, in the more ordinary way, in flakes and little curdled clots; but, instead of rendering the whole milky, the flocculi often incline to a brown colour, looking like the finest particles of bran more or less thickly disseminated throughout the heated urine. Occasionally in these cases the urine has been much loaded with saline ingredients becoming turbid by standing, but rendered quite clear by the application of a much lower degree of heat, than is necessary to coagulate the albumen.

"In all the cases in which I have observed the albuminous urine, it has appeared to me that the kidney has itself acted a more important part, and has been more deranged, both functionally and organically, than has generally been imagined. In the latter class of cases I have always found the kidney decidedly disorganized. In the former, when very recent, I have found the kidney gorged with blood. And in mixed cases, where the attack was recent, although apparently the foundation has been laid for it in a course of intemperance, I have found the kidney likewise disorganized." 4.

Dr. Bright now proceeds to the detail of cases, some of which we shall introduce, under a very abridged form.

*Case* 1. A sailor, aged 34, who, like most sailors, had made free with the grog-bottle, entered Guy's Hospital on the 12th Oct. 1825. He stated that, for the four last years, he had left the sea, and with it the habit of ingurgitation. He was of a pale, unhealthy aspect. Three weeks before admission, he was seized with pains in his loins, knees, and ankles—his legs swelled, and his hands and face were occasionally œdematous. His abdomen, on admission, was painful on pressure—pulse 78—tongue pale—fæces light-coloured—urine scanty, a pint in the 24 hours—appetite good. A pill, containing mercury, squill, and opium, was administered every night, and, during the next five days, he improved, in respect to the urinary secretion; but the œdema was little reduced, and he could not lie easy in bed without being highly propped up. On the 20th of the same month, he was attacked with general inflammatory symptoms in the thorax and abdomen, for which he was bled—had fomentations applied—and took effervescing draughts, with ipecacuan wine. The blood was inflamed. The symptoms returned the next evening, with herpes labialis on the face—and some blood had passed in his motions. The urine, however, was more in quantity, and less sedimentous. On the 25th, the urine was much more free, and it had assumed the dingy brown colour, marking an admixture of red particles of blood. He continued to improve, but complained of *pain and weakness in his loins*. He lies down easily, but his legs continue to swell—tenderness of abdomen gone—urine in good quantity, and clear, but *coagulates by heat*. *27th.* Gums sore from mercury. By the 2d Nov. he was so much improved as to be able to walk about the ward, and was only taking a grain of ipecacuan thrice a day for his bowels. On the evening of the 10th Nov. Mr. Stocker was suddenly called to him for an attack of dyspnœa, with symptoms of thoracic inflammation. *Venesect. ad* ℥x.—*blister.* He was somewhat relieved—the blood was inflamed—but he was quite unable to lie down. The urine again became scanty—and the dyspnœa was increasing. *Squill pill and mercury—venesectio—another blister.* We deem it unnecessary to pursue the diurnal details. The symptoms of thoracic and anasarcous effusion increased, together with the dyspnœa, emaciation, and general prostration. He died on the 29th November.*

*Dissection.* The pericardium contained about four ounces of clear water, which soon became gelatinous. Both portions of pericardium shewed strong marks of inflammation, in the shape of fibrinous deposits, some of recent formation, others of longer standing. The heart was large and firm. The semilunar valves of the aorta shewed ossification. The left lung was every where adherent, and every where converted into "*grey hepatization,*" very few portions admitting air. "The right lung was soft, and in structure not unnatural, but œdematous; filled by the effusion of serum, so that the fluid ran out, mixed with innumerable fine bubbles of air, immediately it was cut into. The whole cavity of the chest, on this side, was filled with serum, but the lung not compressed by it." There was some serous effusion in the abdomen. The peritoneal coat of the liver was coated with a fibrinous deposit, not very recent. No obvious disease in the size or structure of this organ, except that it was—"rather pale-coloured, of a purplish drab throughout, and not of firm consistence." The kidneys were completely granulated throughout, as seen in plate I. with rough external surface, while all traces of natural organization were gone from within, except in the tubular parts, which were of a lighter and more pinky colour than natural.

*Remarks.* Dr. B. thinks that, if we can form any judgment as to the comparative priority of diseased structure in this patient, we should be inclined to give that priority to the disease in the kidney, which "had probably laid the foundation for that effusion into the cellular membrane, which had taken place previously to his admission." Dr. B. observes that there was no

* We see no mention made of auscultation or percussion till the day of this man's death, when the right side of the chest was found to be more sonorous than the left; and, by the assistance of the stethoscope, Dr. B. thought he could hear the heart beat through a fluid. In all cases where thoracic affections are present, the stethoscope should be employed, for we can assure those who cultivate that instrument, that it will require years of study and practice, to make themselves even imperfectly acquainted with the indications which it points out. The dissection of the above case will shew that auscultation, properly employed, would have detected irremediable organic disease in the chest *for months* before the man died.

evidence whatever of organic disease in the liver, anterior to the patient's reception into the hospital—and that it is not at all improbable, that "the greater part of the mischief done to the substance of the left lung, had taken place between the 20th October, when he suffered the severe inflammatory attack, and the 29th November, when he died." The serous effusion was, no doubt, a recent affection.

We are aware that Dr. Bright has the authority of Laennec for the sudden formation of hepatization in the lungs—even the *grey hepatization*, which is the third degree of that disease. With all due deference to M. Laennec, we conceive that, on this point, he may be mistaken. But, granting that hepatization of the lung may take place, say in ten or twenty days, from peripneumony, every pathologist knows that this same hepatization may continue for months or years, without affecting life. And, when we contemplate the state of the above patient when he first came into the hospital, we can have little doubt that hepatization of the lung existed there, and for a very long time before. It is not, therefore, very easy to say, with certainty, that the disorganization of the kidney preceded the hepatization of the lung—and it is still more problematical, that the kidney affection was the cause of the dropsical effusion in the chest. There can be no doubt, however, that the thoracic effusion, especially the inflammation and affection about the heart, was the immediate cause of the fatal termination.

*Case* 2. Eliz. Beaver, aged 37, was admitted on the 23d Nov. 1825, with swelling and fluctuation of the abdomen, attended, also, with tympanitis. The lower extremities, and the parietes of the abdomen were œdematous, with erythema about the ankles. Her face and arms had also swelled occasionally. Severe cough was excited by a deep inspiration, causing, also, some abdominal pain. Her breathing was short—inability to lie horizontally—bad sleep—pulse 112—tongue furred in the middle, and clean at the sides—bowels relaxed—urine clear, but uncertain in quantity. She had been ill about six months, her illness commencing with pain in the chest and increase of cough, to which last she had been subject for four or five years. The catamenia had stopped five months previously. On account of the diarrhœa, confectio opii, and hyd. cum cretâ, were ordered thrice a day, with some other cordial medicines. *24th.* Much the same. On the 25th, the urine was examined, and found to *coagulate by heat*. It was scanty in quantity. She gradually got weaker, and died on the 29th of the same month.

*Dissection.* There was some effusion into both sides of the chest—

body generally anasarcous—lungs tolerably healthy—heart small in size, and feeble in texture, the parietes of the right ventricle being in a state of atrophy—an ounce and a half of water in the pericardium. There was much straw-coloured fluid in the abdomen. The liver, externally, appeared granulated, but this appearance was very much confined to the surface. The kidneys were both of unusual size, and, on external view, they were obviously granulated with yellow matters. The whole of the cortical structure appeared converted into a yellow substance, resembling fat. There was nothing else particular in the dissection.

*Remarks.* Dr. B. thinks we may attribute the dropsy with coagulable urine, in this case, to the disorganization in the kidneys. He seems to doubt whether the state of the heart and liver had any thing to do with the dropsical effusions. There will be some who may doubt this exclusive blame on the kidneys.

In the following cases we shall be more brief in our analysis.

*Case* 3. A female, aged 25, of previous intemperate habits, was admitted Nov. 8th, with anasarcous swelling of the legs, diarrhœa, cough, dyspnœa, bloated and livid face. *The urine was found to coagulate very considerably by heat.* She died on the 12th of January following, after an unsuccessful exhibition of various remedies.

*Dissection.* Nearly two pints of turbid serum in the left side of the chest—lung of that side œdematous and rather fleshy at the summit, with some incipient tuberculation. In the *right* side, there was, also, considerable effusion, and the lung was very much condensed, so that but a small portion admitted air. A thick adventitious membrane surrounded the greater part of it, and it was firmly glued to the pleura. The apex of the right lung was completely tuberculated, with some excavations. The liver was pale, yellowish, rather firm, and inclined to granulation. Ulcerations near the valve of the colon, in the ileum. The kidneys were entirely disorganized. The whole of the cortical substance was of a uniform yellow colour. This state of kidney is beautifully delineated in the second plate.

*Case* 4. A bricklayer, not of intemperate habits, was admitted on the 22d November. Two months previously, having heated himself much in working, he drank cold beer and lay down on the damp grass. His legs began to swell in a day or two afterwards. At the time of admission,he was generally anasarcous, and his legs were greatly swollen, with symptoms of effusion into the cavities. His breathing was much oppressed. Squill pill and mercury, with some other diuretics, were given, with temporary improvement only. His urine was scanty, and *coagulated by heat.* On the 12th December, a diarrhœa, with erysipelas of one leg, came on, and he died on the 16th of the same month.

*Dissection.* Three pints of clear yellow serum in the *right* side of the chest—lung on that side slightly puckered and hardened at the apex. In the *left* side, there was about a pint and a half of serum—left lung healthy. In the right lobe of the liver there was a small collection of tubercular bodies, and a similar collection in the small lobe. "The whole substance of the liver was nearly in a healthy state—a little inclined to be granulated." The cortical structure of the kidneys exhibited the commencement of granulation. They were rather large and soft—general colour pale, and, on stripping off their tunic, the whole surface was seen speckled with minute yellow bodies, which bodies were found pervading the whole cortical substance. These kidneys are delineated in plate the third.

Dr. Bright anticipated this state of kidney before death, and committed the diagnosis to writing.

*Case* 5. A stout looking sailor, aged 34, was admitted on the 29th November. Denied having been intemperate, only taking a good deal of spirits and water. Three years previously, he caught a bad cold, and has never been well since. Five months ago, he began to swell, and his legs and thighs are now decidedly œdematous. The urine is scanty, and *coagulates into a complete gelatinous mass by heat.* Mercury and squills were given, and the urine increased, becoming less coagulable. On the 22d December, dysenteric symptoms came on, and lasted a few days. On the 12th February, we find the urine very scanty, and strongly coagulable. He was evidently declining fast; and now, for the first time, it is stated that "his cough is more troublesome, the expectoration puriform, and, for some days, there have been symptoms of inflammatory affection in the chest." He died on the 14th February.

*Dissection.* Œdema of the lower extremities—considerable effusion into the left cavity of the chest—with flakes of coagulable lymph and other products of inflammation. The lung more firm and red than natural. Nothing wrong in the other side of the chest—heart rather flaccid—liver pale, "inclined to granulation in its appearance, but not enlarged, nor materially firmer than natural." Unequivocal evidences of peritoneal inflammation were observable, with considerable effusion. The kidneys were large—very dark on their upper surface, and mottled with yellow on their under surface. Internally, the structure had changed to a fatty substance, with some traces of granulation.

The foregoing half dozen of cases out of 25 put on record by Dr. Bright, will be sufficient specimens for this analysis; and we shall, therefore, proceed to give some account of our author's "GENERAL OBSERVATIONS" appended to the narrative of facts.

From the facts which have come under Dr. B's notice, he thinks he is authorised to establish three varieties, if not three completely separate forms, of diseased structure in the kidneys —generally attended by a decidedly albuminous character in the urine.

" In the *first*, a state of degeneracy seems to exist, which from its appearance might be regarded as marking little more than simple debility of the organ. In this case the kidney loses its usual firmness, becomes of a yellow mottled appearance externally; and when a section is made, nearly the same yellow colour slightly tinged with gray is seen to pervade the whole of the cortical part, and the tubular portions are of a lighter colour than natural. The size of the kidney is not materially altered, nor is there any obvious morbid deposit to be discovered. (Plate II. Fig. 4.) This state of the organ is sometimes connected with a cachectic condition of body, attended with chronic disease, where no dropsical effusion has taken place either into the cellular membrane or into the cavities of the body; I have found it in a case of diarrhœa and phthisis, and in a case of ovarian tumour. In the former it was connected with slight and almost doubtful coagulation of the urine by heat; in the latter I had omitted to examine the state of the urine. I also met with nearly the same condition of the kidney, with some opake yellow deposits interspersed through the structure, in the case of a man who died exhausted with diarrhœa brought on by hardships and intemperance, and in whose case the secretion of urine was very deficient, but whether coagulable or not I had no opportunity of ascertaining. When this disease has gone to its utmost, it has appeared to terminate by producing a more decided alteration in the structure; some portions becoming consolidated, so as to admit of very partial circulation; in which state the surface has assumed a somewhat tuberculated appearance, the gentle projections of which were paler than the rest, and scarcely received any of the injection which was thrown in by the arteries. (Plate II. Fig. 1. 2. and 3.) In this more advanced stage, if it be the same disease, dropsy has existed, and the urine has been coagulable." 67.

The *second* form, is that in which the whole cortical part is converted into a granulated texture, and where there appears to be a copious morbid interstitial deposit of an opake white substance. In the early stage, when the tunic is taken off, there is exhibited only an increase of the natural fine mottled appearance given by the healthy structure of the kidney. On slitting the organ longitudinally, a slight appearance of the same kind is discovered internally, and the kidney is deficient in its natural firmness. In time, the deposited matter becomes more abundant and is seen in numerous specks of no definite form, thickly strewed on the surface. Internally, these specks are found distributed in a more or less regular manner throughout the whole cortical substance, no longer presenting a doubtful appearance, but manifest to the eye without any preparation. At a still more advanced period, the granulated texture begins to shew itself externally, in slight uneven projections on the surface of the kidney, very apparent through the tunic. The organ is generally larger than natural, sometimes not at all increased in size.

"The *third* form of disease is where the kidney is quite rough and scabrous to the touch externally, and is seen to rise in numerous projections not much exceeding a large pin's head, yellow, red, and purplish. The form of the kidney is often inclined to be lobulated, the feel is hard, and on making an incision the texture is found approaching to semicartilaginous firmness, giving great resistance to the knife. The tubular portions are observed to be drawn near to the surface of the kidney: it appears in short like a contraction of every part of the organ, with less interstitial deposit than in the last variety. This form of disease existed in a case from which I had a drawing executed about three years ago, it also existed in Bonham, (p. 22.); and a most decidedly marked instance of it may be found in Stewart, (Plate III. Fig. 1 and 2,) where however the kidney was of a lighter colour than in the other cases, which were more of a purplish gray tinge. I believe the case of Smith, (p. 23,) belonged to the same. In most of these cases the urine has been highly coagulable by heat, at times forming a large curdled deposit, though in one case (Castles) where an approach to this appearance was found on the outside of the kidney, but with marked structural change in the liver, and with confirmed bronchial congestion, only a dense bran-like deposit of a brown colour was produced by the application of heat." 69.

Although Dr. B. hazards a conjecture as to the existence of these three different forms, he is by no means confident as to the correctness of this view. So much for the descriptive part. We now come to—

### Observations on the Treatment.

It has been our author's object, in all that precedes, to prove that certain dropsical affections depend more on derangement of the kidneys themselves, than has generally been supposed—and that the particular cases in which these organs are the seat of disease, are pointed out by the albuminous nature of the urine. The author wishes he could add any thing very satisfactory as to the treatment. But he is inclined to doubt whether it be possible to effect a cure, or even afford much relief after the decided organic change has taken a firm hold on the kidney. In sudden attacks of anasarca from intemperance and exposure, in the early stage, and before organic changes have taken place, we have *first* to restore the healthy action of the kidney—and, *secondly*, to guard against those dangerous secondary consequences which may destroy the patient at any period of the disease. Inflammatory affections, especially of the serous membranes, and serous or sanguineous effusions on the brain, are the two principal sources of danger. Thus, out of seventeen dissections, they found ten or eleven betraying pleural inflammation, ancient or recent—five of pericardial phlogosis, (three recent, two old,) and only one where peritoneal inflammation was well marked. In respect to cerebral affections supervening on renal disease, the cases recorded by our author present both apoplexy and epilepsy. The treatment must, therefore, bear on the prevention of these impending dangers, and active depletion, in the early stages, is indispensibly necessary. When symptoms indicative of the presence of these inflammatory affections appear, there can no longer be a doubt as to the free abstraction of blood. Practitioners should bear in mind that, in these complaints, the thoracic inflammations are extremely insidious, and are often masked by the hydropic phenomena. "And we are led to ascribe many of the symptoms—such as the slight cough, the dyspnœa, and the difficulty of lying down—to effusion rather than inflammation." We are sorry to hear such observations from Dr. Bright, at a period when effusion in the chest may be very readily distinguished from inflammation of the lungs or pleura, by even tyros in auscultation.

When the inflammatory attack comes on early in the disease, it is often overcome by free depletion; but in the more advanced stages, the patient bears depletion so ill as to check the depletory measures. But bleeding is also an important remedy for the restoration of healthy action in the kidneys themselves. The foundation of future disorganization is probably laid in a previous state of slow inflammation or congestion. General blood-letting was useful in many cases—in others, local depletion from the loins had a better effect.

Purgatives, especially the saline laxatives combining diuretic powers, are decidedly beneficial. The supertartrate of potash was found very useful in our author's hands. He gave it in a fully saturated solution—a large draught early in the morning. The next diuretic which Dr. B. has been in the habit of employing, was squill, in its various preparations—especially when combined with hyosciamus or opium. Digitalis, where the pulse was sharp, seemed adapted to the complaint. When the inflammatory stage had subsided, Dr. B. thought he saw advantage derived from turpentine and Peruvian balsam.

In respect to the employment of mercury in this class of diseases, Dr. Bright seems to be of opinion that it is injurious, rather than advantageous; although it is consistent with good and successful practice in most other inflammations to avail ourselves of the valuable combination of calomel and opium. Still Dr. B. appears to be in doubt upon this subject. He observes, however, that the sphere of mercurial practice, in these diseases, is very much limited, on account of the rapidity with which ptyalism comes on, and the difficulty of restraining it afterwards. When the cellular membrane is anasarcous, from renal disorganization, the gums and cheeks are not capable of supporting the process of ulceration, and often pass into a state of gangrene.

"Where, as in a case to which I have only referred, we have a flaccid, watery and dissolved state of the kidney, I can point out no diagnostic symptoms by which it can be discovered, except such as show general debility of circulation and feebleness in the structure of the heart; for probably the feeble condition of the two organs may often be found co-existent. If this be the case, it is not improbable that Tonics will be the most appropriate remedies. In one or two cases of anasarca which I have lately had under my care, where from the feeble but extensive beat of the heart I was led to suppose that a feeble state of that organ existed, a combination of Sulphate of Quinine with Squill, effectually restored the patient. And occasionally we find anasarca even with coagulable urine so marked by debility, that tonics and steel give decided relief; probably it is as a tonic that the Uva Ursi is sometimes useful." 74.

### II. CHEMICAL PROPERTIES OF THE URINE.

Dr. Bostock has favoured the author with a letter on this subject, from which we shall extract some particulars. In the greater number of specimens of urine examined by Dr. Bostock, as passed by the patients whose cases are narrated, the quantity of matter dissolved or suspended was below the average of healthy urine. Dr. B.'s experiments induce him to conclude, that these specimens of urine were not only deficient in some of the natural constituents, but contained a quantity of extraneous matters. The coagulability of the dropsical urine, Dr. Bostock attributes to the presence of albumen; but thinks that this proximate principle is modified or altered, in some cases of the disease under consideration.

Here Dr. B. trenches a little on the pathological physician's province. He observes that the presence of albumen is commonly considered a morbid phenomenon, and a pathognomonic symptom of a certain state of the constitution, or, indeed, of a specific disease. If the albumen be in a state coagulable by heat, the first position may be true; "but it must be admitted, on the other hand, that an albuminous state of the urine is produced by such a variety of circumstances, and many of them of so trifling a nature, as to render it almost a constant occurrence." In his own person, he has hardly ever found the urine entirely free from albumen, and he observed it to be increased to a considerable amount by the slightest causes.

This brings our analysis of the first part of Dr. Bright's work, occupying 88 quarto pages, to a close. In our next number, we shall pursue our analysis, so as to make our readers as well acquainted with the work as can be done through the medium of a journal, and without the assistance of the plates. We strenuously recommend again this very meritorious production to the patronage of the affluent members of our profession, and think that no medical society or association should be without it.

